# Cold stress in rice (*Oryza sativa* L.): Molecular mechanisms of sensing, signaling, transcriptional regulation, membrane lipid remodeling, and hormonal modulation

**DOI:** 10.3389/fpls.2025.1729934

**Published:** 2026-01-05

**Authors:** Peixiang Xiao, Meixin Xiong, Kexin Hou, Xueyan Guo, Hua Li, Yi Liu

**Affiliations:** 1 Jiangxi Key Laboratory for Sustainable Utilization of Chinese Materia Medica Resources, Lushan Botanical Garden, Jiangxi Province and Chinese Academy of Sciences, Jiujiang, Jiangxi, China; 2 Lushan Botanical Garden, Jiangxi Province and Chinese Academy of Sciences, Jiujiang, Jiangxi, China; 3 College of Horticulture, South China Agricultural University, Guangzhou, Guangdong, China

**Keywords:** cold stress, membrane lipid remodeling, molecular mechanism, phytohormone, rice (*Oryza sativa* L.), signaling transduction, transcriptional regulation

## Abstract

Rice (*Oryza sativa* L.) is a staple crop. It was originally domesticated in tropical and subtropical regions, sustains nearly half of the global population and contributes approximately 20% of the world’s total dietary energy supply. However, its inherent sensitivity to low-temperature severely threatens yield stability. To meet the growing global food demand, rice cultivation is expanding to low-temperature-prone high-altitude and high-latitude regions. This expansion makes the low-temperature sensitivity problem worse. To cope with cold stress, rice has evolved a sophisticated regulatory network for cold sensing, signal transduction, and response. Recent research progress includes identifying key sensors (COLD1-RGA1, COG1-OsSERL2), characterizing secondary messengers (Ca²^+^, 2’,3’-cAMP, ROS) and downstream cascades (CBL-CIPK, CDPK, MAPK), elucidating core transcriptional modules (OsbHLH002/OsICE1-OsDREBs-COR) and auxiliary transcriptional factors (WRKY, MYB, NAC), uncovering critical genes involved in membrane lipid remodeling, and defining the roles of phytohormones (ABA and GA) that fine-tune cold stress responses. This review summarizes current understanding of these molecular mechanisms and highlights future directions for rice cold stress research.

## 1 Introduction

Rice (*Oryza sativa* L.), as a cornerstone of global food systems, is ranked among the most indispensable staple food crops worldwide. Beyond serving as a dietary mainstay, rice sustains nearly half of the global population. Dependence on rice is particularly high in densely populated regions of Asia, Africa, and Latin America. Rice contributes approximately 20% of the world’s total dietary energy supply. This proportion exceeds 70% in major rice-consuming countries of Asian (Bin Rahman and Zhang, 2022). Global cultivation and production data of rice underscore its great importance. In 2019, the global rice cultivation area reached around 161 million hectares, with a total production of 755 million tons (Bin Rahman and Zhang, 2022). However, this production scale is under increasingly pressure. Multiple challenges are driving this pressure, with the rapid growth of the global population being the most significant one (Yuan et al., 2021). Against this backdrop, ensuring long-term food security has become a pressing global priority. Consequently, expanding the boundaries of rice cultivation has emerged as a critical strategic imperative.

A key approach to addressing this challenge lies in extending rice cultivation into high-altitude and high-latitude regions. Historically, these regions were deemed unsuitable for rice growth due to their inherent low-temperature conditions during rice growing period. Yet, rice is a thermophilic crop that originated and was domesticated in the warm, humid tropical and subtropical regions (Kovach et al., 2007; Huang et al., 2012). Over millennia of domestication and selection, its genetic makeup has been optimized for warm environments. Therefore, cold stress is one of the primary abiotic stresses causing severe rice yield losses (Cruz et al., 2013).

In general, cold stress refers to temperatures below an optimum threshold. These temperatures are low enough to inhibit plant growth. For plant cultivation, cold stress can be categorized into three distinct temperature zones. The first is freezing conditions, which are below 0°C. The second is chilling conditions. They span from ≥ 0°C to the plant’s growth limit. The third is suboptimum conditions. They range from the growth limit up to the optimal temperature (Soualiou et al., 2022). The severity of cold-induced damage to rice depends on three interrelated critical factors, including the rice growth stage, the magnitude of low-temperature exposure, and the duration of cold stress persistence. Morphologically, typical cold damage phenotypes include delayed seed germination, inhibited vegetative growth, reduced tiller number, leaf curling and wilting, impaired fertilization, reduced grain filling efficiency. In extreme cases, seedling death occurs (Li et al., 2022; Soualiou et al., 2022; Yang, 2022). These multifaceted impacts highlight the urgency of understanding how rice responds to cold stress and developing cold-tolerant varieties.

To cope with low-temperature conditions, rice has evolved a sophisticated and coordinated regulatory network that encompasses three core processes: cold stress perception, signal transduction, and the activation of stress-responsive reactions (Li et al., 2022; Ding et al., 2024). This network involves a suite of molecular components, including membrane-localized cold sensors, secondary messengers, transcription factors, and downstream functional genes (Li et al., 2022; Ding et al., 2024). Understanding the molecular mechanisms of this signaling network is not only fundamental to advancing our knowledge of plant stress biology in rice but also essential for developing cold-tolerant rice varieties through molecular breeding or genetic engineering. Such efforts are critical for stabilizing rice production in existing cold-prone regions and unlocking the agricultural potential of high-altitude and high-latitude regions. In recent years, advances in molecular biology techniques, including high-throughput sequencing, transcriptomics, proteomics, and gene editing (e.g., CRISPR-Cas9), have accelerated research in rice cold stress. Extensive studies have identified numerous important genes and regulatory factors involved in cold tolerance (Li et al., 2022; Ding et al., 2024). This review aims to systematically summarize the advances in understanding cold stress sensing, signaling, transcriptional regulation, and hormonal modulation in rice. Additionally, we will outline prospective directions for future research which may provide a roadmap for advancing rice cold stress research and addressing the challenges of global food security.

## 2 Sensors and secondary messengers of cold stress in rice

### 2.1 Cold sensors

#### 2.1.1 The COLD1-RGA1 complex

In rice, the initial perception of cold stress and the subsequent activation of signaling pathways occur within an extremely short timeframe (minutes) after exposure to low temperatures. This short timeframe brings significant challenges for identifying cold sensors and investigating early-stage signal transduction.

**Figure 1:**
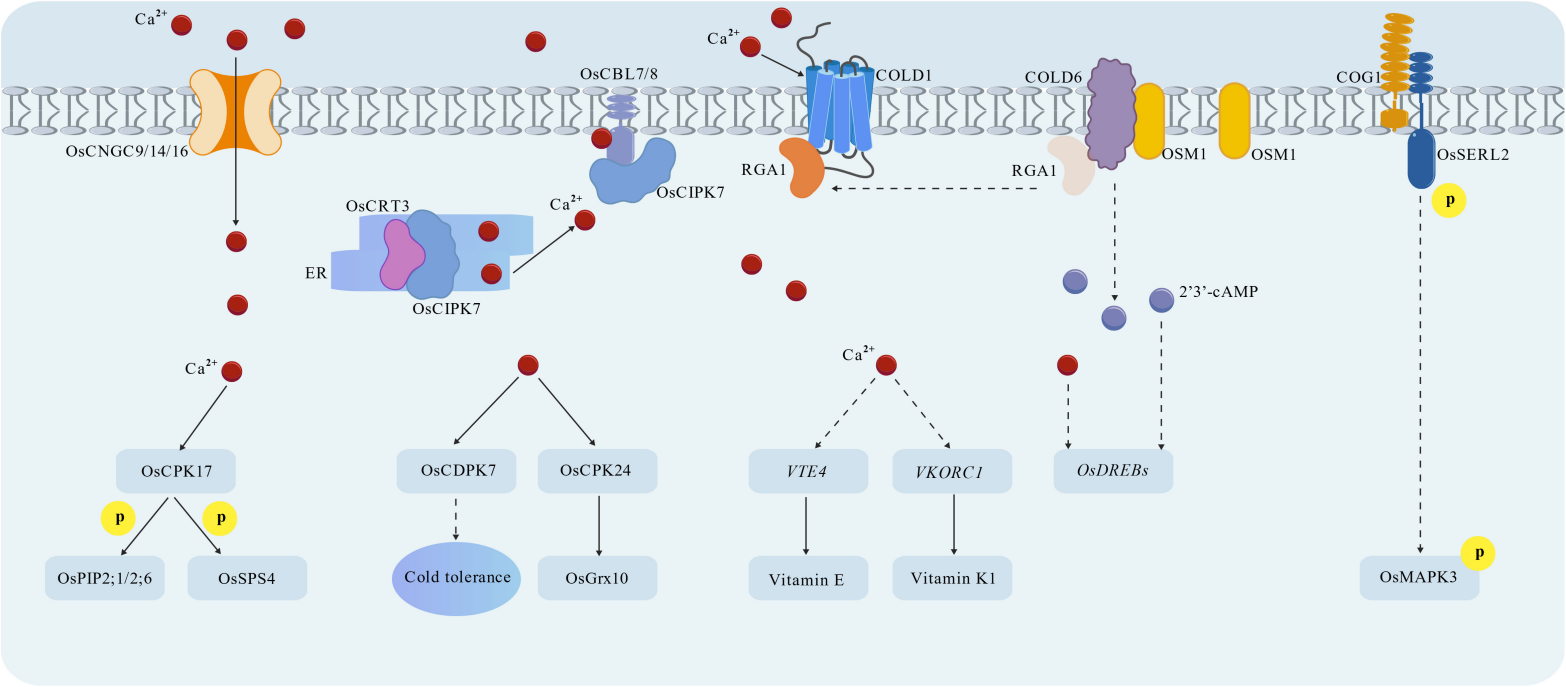
Sensors and secondary messengers of cold stress in rice.

After decades of intensive research, the COLD1-RGA1 complex was identified as a pivotal cold sensor in rice (**Figure 1**) (Ma et al., 2015). COLD1 is localized to both the plasma membrane and endoplasmic reticulum (ER). Under cold stress, it interacts with the G-protein α subunit (RGA1) to activate Ca²^+^ channels, which trigger the release of Ca²^+^, a key secondary messenger, into the cytoplasm (Ma et al., 2015). This cytoplasmic Ca²^+^ influx further modulates downstream signal transduction and stress responses. Notably, one critical outcome of this Ca²^+^ influx is that it regulates the contents of Vitamin E and Vitamin K1 to alleviate cold stress by mediating tocopherol O-methyltransferase (*VTE4*) and vitamin K epoxide reductase complex subunit 1 (*VKORC1*), respectively (Luo et al., 2021). Map-based cloning studies have demonstrated that the cold-resistant allelic variant *COLD1*^jap^ (derived from *japonica* rice) enables rice adaptation to cold stress. A single nucleotide polymorphism (SNP) in the fourth exon of *COLD1*^jap^, designated SNP2, is responsible for this cold tolerance. An A-to-T/C transition at the SNP2 locus distinguishes *japonica* from *indica* rice cultivars: the ancestral A allele encodes Lysine (K187) in *japonica* rice, whereas the T/C allele encodes either Methionine (M187) or Threonine (T187) in *indica* rice (**Table 1**) (Ma et al., 2015). Phylogenetic and evolutionary analyses revealed that SNP2 originated from Chinese wild rice (*O. rufipogon*) and was selected during the domestication of *japonica* rice (Ma et al., 2015).

**Table 1:** Genes/QTLs in rice cold stress responses.

Genes/QTLs	LOC number	Growth Stage	Field validation	Reference
*COLD1*	LOC_Os04g51180	V	No	(Ma et al., 2015)
*RGA1*	LOC_Os05g26890	V	No	(Ma et al., 2015) (Luo et al., 2024)
*VTE4*	LOC_Os02g47310	V	No	(Luo et al., 2021)
*VKORC1*	LOC_Os03g03949	V	No	
*COG1*	LOC_Os11g36200	V	No	(Xia et al., 2023)
*OsSERL2*	LOC_Os06g16330	V	No	
*OsMAPK3*	LOC_Os03g17700	V	No	(Xia et al., 2023) (Zhang et al., 2017) (Xia et al., 2021)
*OsCNGC14*	LOC_Os03g55100	V	No	(Cui et al., 2020)
*OsCNGC16*	LOC_Os05g42250	V	No	
*OsCNGC9*	LOC_Os09g38580	V	No	(Wang et al., 2021) (Wu et al., 2024)
*COLD6*	LOC_Os06g04130	V	No	(Luo et al., 2024)
*OSM1*	LOC_Os12g38170	V	No	
*OsCRT3*	LOC_Os01g67054	V	No	(Guo et al., 2023)
*OsCIPK7*	LOC_Os03g43440	V	No	(Zhang et al., 2019a) (Guo et al., 2023)
*OsCPK17*	LOC_Os07g06740	V/R	No	(Almadanim et al., 2017)
*OsPIP2;1*	LOC_Os07g26690	V	No	
*OsPIP2;6*	LOC_Os04g16450	V	No	
*OsSPS4*	LOC_Os08g20660	V	No	
*OsCPK24*	LOC_Os11g07040	G/V/R	No	(Liu et al., 2018b)
*OsGrx10*	LOC_Os02g43180	V	No	
*OsCDPK7*	LOC_Os04g49510	V	No	(Saijo et al., 2000)
*HDA705*	LOC_Os08g25570	V	No	(Ma et al., 2025)
*OsECT3*	LOC_Os03g06240	V	No	
*OsHDA716*	LOC_Os05g36930	V/R	No	(Sun et al., 2024) (Kamble, 2024)
*OsbZIP46*	LOC_Os06g10880	V/R	No	
*OsDREB1A*	LOC_Os09g35030	V	No	(Lourenco et al., 2013) (Wang et al., 2021)
*OsSRO1c*	LOC_Os03g12820	V/R	Yes	(Hu et al., 2024)
*OsDREB2B*	LOC_Os05g27930	V	No	
*OsbHLH002/OsICE1*	LOC_Os11g32100	V	No	(Nakamura et al., 2011) (Zhai et al., 2024) (Zhang et al., 2017) (Liu et al., 2023a) (Xia et al., 2021)
*OsEIN2*	LOC_Os07g06130	V	No	(Zhai et al., 2024)
*OsEIL1*	LOC_Os03g20790	V	No	
*OsEIL2*	LOC_Os07g48630	V	No	
*OsHOS1*	LOC_Os03g52700	V	No	(Lourenco et al., 2013)
*OsMAPK6*	LOC_Os06g06090	V	No	(Liu et al., 2023a)
*OsTPP1*	LOC_Os02g44230	V	Yes	(Nakamura et al., 2011) (Zhang et al., 2017) (Yu et al., 2024)
*OsPP2C27*	LOC_Os02g55560	V	No	(Xia et al., 2021)
*IPA1*	LOC_Os08g39890	V	No	(Liu et al., 2023a) (Jia et al., 2022)
*OsSAPK9*	LOC_ Os12g39630	V	No	(Xu et al., 2024)
*OsERF52*	LOC_Os05g49700	V/R	Yes	
*OsSAPK6*	LOC_Os02g34600	V	No	(Jia et al., 2022)
*OsSAPK8*	LOC_Os03g55600	V	No	(Jia et al., 2022) (Wang et al., 2021)
*OsCTK1*	LOC_Os01g13060	V	No	(Wu et al., 2024)
*OsP3B*	LOC_Os01g13080	V	No	
*OsMKP1*	LOC_Os05g02500	V	No	
*OsTPS2*	LOC_Os01g 54560	V	No	(Shu et al., 2023) (Li et al., 2024a)
*OsMYB30*	LOC_Os02g41510	G/V	Yes	(Yu et al., 2024)
*ABF1*	LOC_Os01g64730	V	No	(Shu et al., 2023)
*OsSAPK10*	LOC_Os03g41460	V	No	
*OsCHR11*	LOC_Os01g27040	V	No	(Li et al., 2024a)
*OsCHR17*	LOC_Os05g05780	V	No	
*OsWRKY63*	LOC_Os11g45920	V/R	Yes	(Zhang et al., 2022)
*OsWRKY76*	LOC_Os09g25060	V	No	
*OsbHLH148*	LOC_Os03g53020	V	No	(Zhang et al., 2022) (Zhu et al., 2024)
*MYBS3*	LOC_Os10g41200	V	Yes	(Zhang et al., 2022) (Zhu et al., 2024) (Su et al., 2010)
*OsTTG1*	LOC_Os02g45810	V	No	(Zhu et al., 2024)
*OsDOF1*	LOC_Os01g09720	R,	No	(Liu et al., 2021) (Liu et al., 2023b)
*OsZOS2-19*	LOC_Os02g57790	V	No	(Zhang et al., 2025)
*OsPGL12*	LOC_Os12g10184	V/R	No	(Chen et al., 2018)
*OsWRKY71*	LOC_Os02g08440	V	No	(Kim et al., 2016) (Li et al., 2025).
*OsMYB2*	LOC_Os3g20090	V	No	(Zhou and Tang, 2019)
*OsCycB1;1*	LOC_Os01g59120	V	No	(Zhou and Tang, 2019)
*OsCycB2;1*	LOC_Os04g47580	V	No	
*OsMYB3R-2*	LOC_Os01g62410	V	No	(Zhou and Tang, 2019)
*OsWRKY53*	LOC_Os05g27730	R	Yes	(Tang et al., 2022)
*GA20ox1*	LOC_Os03g63970	V/R	No	(Tang et al., 2022) (Li et al., 2021)
*GA20ox3*	LOC_Os07g07420	R	No	(Tang et al., 2022)
*GA3ox1*	LOC_Os05g08540	R	No	
*UDT1*	LOC_Os07g36460	R	No	
*TDR*	LOC_Os02g02820	R	No	
*SLR1*	LOC_Os03 g49990	V/R	No	(Tang et al., 2022) (Li et al., 2021)
*OsGRF6*	LOC_Os03g51970	V	No	(Li et al., 2021)
*CTB5*	LOC_Os07g39320	V/R	Yes	(Guo et al., 2025)
*OsHox12*	LOC_Os03g10210	R	Yes	
*OsGA2ox6*	LOC_Os04g44150	R	Yes	
*OsNCED3*	LOC_Os03g44380	V	No	(Li et al., 2025) (Xiang et al., 2024) (Liu et al., 2023b)
*OsNCED4*	LOC_Os07g05940	V	No	(Xiang et al., 2024)
*OsNCED5*	LOC_Os12g42280	V	No	(Xiang et al., 2025) (Li et al., 2025)
*OsNAC5*	LOC_Os11g08210	G/V	No	(Li et al., 2024b)
*OsABI5*	LOC_Os01g64000	G/V	No	
*OsPP2C30*	LOC_Os03g16170	V	No	
*OsPRX70*	LOC_Os05g04490	V	No	
*bZIP73*	LOC_Os09g29820	V/R	Yes	(Liu et al., 2018a) (Liu et al., 2019)
*bZIP71*	LOC_Os09g13570	V/R	No	
*qLTG3-1*	LOC_Os03g01320	G/R	Yes	(Liu et al., 2019)
*OsABA8ox1*	LOC_Os02g47470	V	No	(Li et al., 2025)
*OsDREB1B*	LOC_Os09g35010	V	No	(Nakamura et al., 2011) (Lourenco et al., 2013) (Zhang et al., 2022)
*OsFAD8*	LOC_Os07g49310	V	Yes	(Tovuu et al., 2016)
*OsTIL1*	LOC_Os02g39930	V	No	(Ji et al., 2024).
*OsFAD3-1*	LOC_Os11g01340	V	No	
*OsFAD3-2*	LOC_Os12g01370	V	No	
*OsFAD7*	LOC_Os03g18070	V	No	
*CTB6*	Os10g11730	R	Yes	(Gao et al., 2025)
*OsKASI-2*	LOC_Os04g36800	V/R	No	(Zhang et al., 2024)
*OsPLDα1*	LOC_Os01g07760	G/V	No	(Huo et al., 2016)
*OsPUS1*	LOC_Os03g05806	V	No	(Wang et al., 2022)
*SOP10*	LOC_Os02g07050	V	No	(Wang et al., 2022) (Zu et al., 2023)

#### 2.1.2 The COG1-OsSERL2 complex

In addition to the COLD1-RGA1 complex, the COG1-OsSERL2 complex represents another cold sensor that initiates a distinct signaling cascade in *japonica* rice (**Figure 1**) (Xia et al., 2023). COG1 is a membrane-localized leucine-rich repeat receptor-like protein (LRR-RLP). Under cold stress, COG1 associates with and activates OsSERL2, a plasma membrane-localized kinase. The cold signal perceived by the COG1-OsSERL2 complex then activates mitogen-activated protein kinase 3 (OsMAPK3) in the cytoplasm, ultimately enhancing cold tolerance (**Table 1**) (Xia et al., 2023). Similar to *COLD1*^jap^, the functional *COG1* allele also originated from *O. rufipogon* and was fixed in *japonica* rice during domestication. This highlights the role of wild rice germplasm in the evolution of cold tolerance in cultivated rice (Xia et al., 2023).

### 2.2 Secondary messengers in Cold signaling

Ca²^+^ is widely recognized as the primary and canonical secondary messenger in rice cold stress signaling pathways (**Figure 1**) (Ding et al., 2019). The cold-induced cytoplasmic Ca²^+^ influx (triggered by the COLD1-RGA1 complex) is mainly mediated by cyclic nucleotide-gated channels (CNGCs), including OsCNGC9, OsCNGC14, and OsCNGC16 (Cui et al., 2020; Wang et al., 2021). Additionally, the endoplasmic reticulum (ER)-localized Ca²^+^-binding protein calreticulin (OsCRT3) contributes to the cold-induced elevation of cytosolic Ca²^+^ concentrations (**Table 1**) (Guo et al., 2023).

Recent studies have identified 2’,3’-cyclic adenosine monophosphate (2’,3’-cAMP) as a crucial secondary messenger in rice cold signaling and other biotic stress signaling (**Figure 1**) (Yu et al., 2022; Luo et al., 2024). Under normal temperature conditions, COLD6 interacts with RGA1 at the plasma membrane. Upon exposure to cold stress, cold-induced osmotin-like protein 1 (OSM1) binds to COLD6, displacing RGA1 from the COLD6-RGA1 complex. This displacement triggers an increase in intracellular 2’,3’-cAMP levels, which in turn enhances chilling tolerance (Luo et al., 2024). Functional analyses have shown that COLD6 negatively regulates cold tolerance and depends on OSM1 for its function in cold stress responses. Both the accumulation of OSM1 and the interaction between COLD6 and OSM1 significantly elevate 2’,3’-cAMP concentrations, which further activate the expression of cold-responsive (COR) genes, such as members of the dehydration-responsive element-binding protein (OsDREB) family (Dubouzet et al., 2003; Ito et al., 2006; Luo et al., 2024). Collectively, OSM1 competes with RGA1 for binding to COLD6. Under cold stress, RGA1 is released from the COLD6-RGA1 complex and subsequently interacts with COLD1 to induce Ca²^+^ influx, while free COLD6 binds to OSM1 to trigger 2’,3’-cAMP accumulation (**Table 1**) (Ma et al., 2015; Luo et al., 2024). Notably, this coordinated interplay ensures the integration of Ca²^+^ and 2’,3’-cAMP signaling pathways in response to cold stress.

Reactive oxygen species (ROS) also function as secondary messengers in rice responses to cold stress, as well as to numerous other abiotic and biotic stresses (Waszczak et al., 2018; Sinenko et al., 2021; Mittler et al., 2022; Wu et al., 2023; Sood, 2025; Zheng et al., 2025). ROS encompass superoxide ion (O_2_^•–^), hydrogen peroxide (H_2_O_2_), singlet oxygen (^1^O_2_), ozone, hydroxyl radical (OH^•^), and organic and inorganic peroxides, which play pivotal role in initiating plant defense responses against various stresses (Mittler et al., 2022). However, excessive accumulation of ROS can induce substantial cellular damage by triggering the overoxidation of essential macromolecules, including lipids, amino acids and DNA. To ensure normal growth and stress resilience, ROS homeostasis is tightly regulated by a dynamic balance between ROS production and scavenging systems (Waszczak et al., 2018; Mittler et al., 2022). It is well established that cold stress induces ROS accumulation in rice (Li et al., 2022). Wang et al. demonstrated that OsPUS1, a pseudouridine synthase, is critical for maintaining ROS homeostasis in rice under cold stress (Wang et al., 2022); *ospus1* mutants exhibit overproduction of O_2_^•–^ from the mitochondrial complex 1 and display albino seedlings at low temperature (Wang et al., 2022; Zu et al., 2023). Further investigations revealed that SOP10, a mitochondrial pentatricopeptide repeat protein, contributes to overproduction of O_2_^•–^in *ospus1* mutants, and knockout of *SOP10* can rescue the albino phenotype of *ospus1* under cold stress (Zu et al., 2023). Additional evidence from the same study indicated that mitochondrial superoxide is essential for rice cold stress responses, as overexpression of superoxide-scavenging enzymes can enhance rice cold tolerance (Zu et al., 2023). Although these studies have uncovered certain mechanism underlying ROS accumulation and scavenging in rice cold stress responses, more efforts are required to fully elucidate the entire regulatory network maintaining ROS homeostasis under cold stress.

### 2.3 Decoding Ca²^+^ signals

In addition to regulate the cytosolic Ca^2+^ concentration (Guo et al., 2023), OsCRT3 is also involved in Ca^2+^ signal decoding process (**Figure 1**). Under normal temperatures, OsCRT3 weakly interacts with OsCIPK7 (a CBL-interacting protein kinase), and the kinase activity of OsCIPK7 is repressed by its auto-inhibitory domain (Weinl and Kudla, 2009). When cold stress occurs, OsCRT3 undergoes a conformational change that strengthens its interaction with OsCIPK7, boosting the kinase activity of OsCIPK7. Moreover, the elevated cytosolic Ca²^+^ is bound and perceived by calmodulin-like proteins (OsCBL7 and OsCBL8), which then physically interact with OsCIPK7 at the plasma membrane to further activate the kinase activity of OsCIPK7, thereby promoting cold tolerance in rice (Guo et al., 2023). The critical role of OsCIPK7 in cold tolerance is further supported by the genetic evidence that a point mutation which increases the kinase activity of OsCIPK7 results in enhanced cold tolerance in rice plants (Zhang et al., 2019a).

Beyond OsCBL7, OsCBL8, and OsCIPK7, calcium-dependent protein kinases (CDPKs) also play essential roles in decoding Ca²^+^ signals during rice cold stress responses (**Figure 1**). The fine-tuned regulation of OsCPK17 (a CDPK family member) is critical for rice cold tolerance. Both knockout and overexpression of OsCPK17 render rice more sensitive to cold stress, indicating that the accumulation of OsCPK17 must be maintained within a narrow optimal range (Almadanim et al., 2017). Activated OsCPK17 phosphorylates target proteins including aquaporins (OsPIP1;2, OsPIP2;6) and sucrose-phosphate synthase (OsSPS4), which are hypothesized to contribute to cold tolerance by regulating water homeostasis and sugar metabolism, respectively (Almadanim et al., 2017). Another CDPK, OsCPK24, is induced by cold stress and acts as a positive regulator of cold tolerance (Liu et al., 2018b). Mechanistically, OsCPK24 phosphorylates OsGrx10, a glutathione-dependent thioltransferase, and inhibits the thioltransferase activity of OsGrx10. This inhibition maintains higher intracellular reduced glutathione levels, which enhances redox homeostasis and protects cells from cold-induced oxidative damage (Liu et al., 2018b). OsCDPK7, a third key CDPK, is also induced by cold stress and functions as a positive regulator of cold tolerance, though its specific downstream targets and detailed mechanism of action remain to be fully elucidated (Saijo et al., 2000).

### 2.4 Transcriptional regulation of *COLD1*

**Figure 2:**
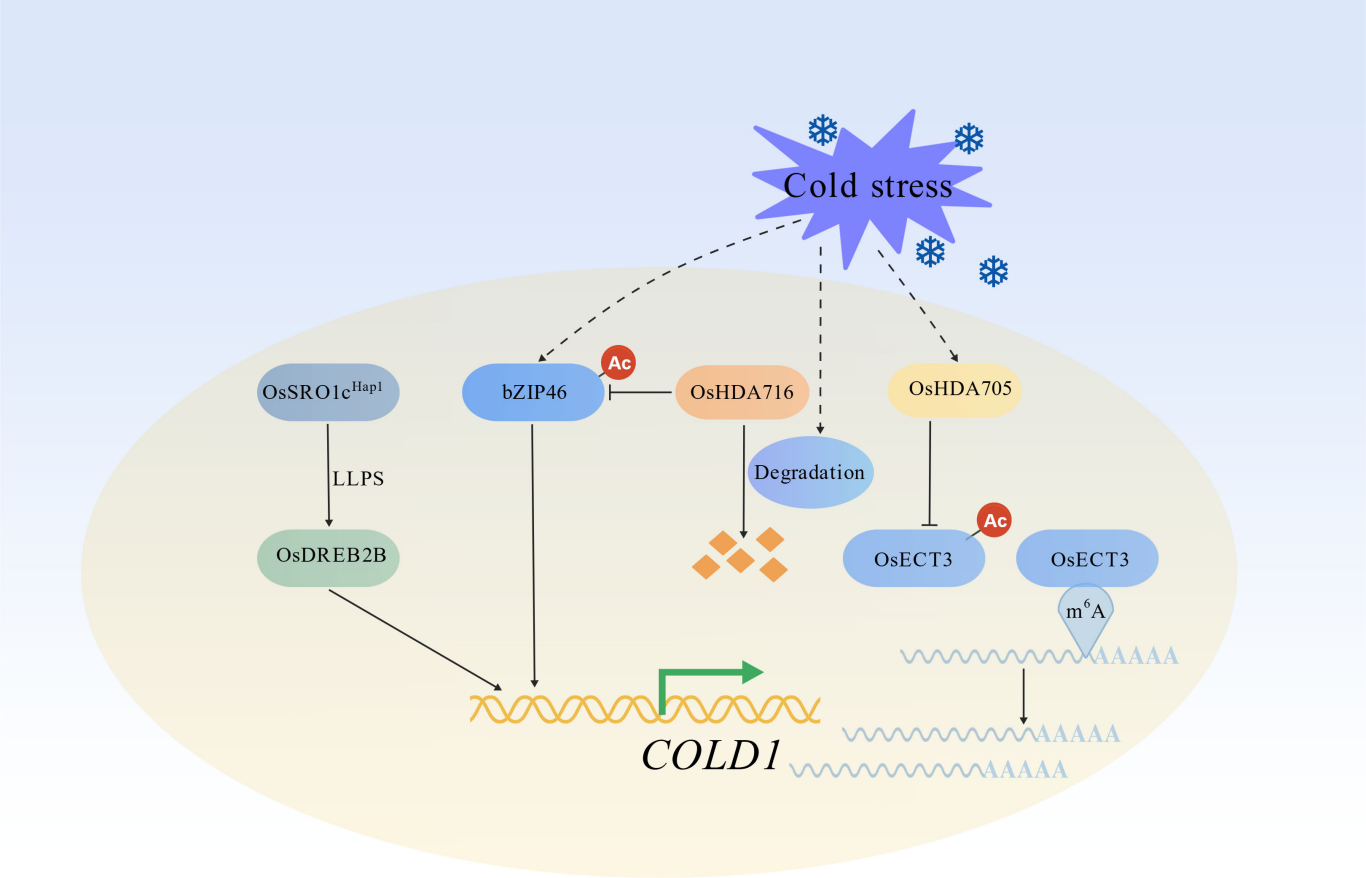
Transcriptional regulation of *COLD1*.

As a central cold sensor in rice, the transcriptional regulation of *COLD1* has been extensively studied (**Figure 2**). Histone deacetylases (HDACs) have emerged as critical regulators of *COLD1* expression. Under cold stress, cold-induced histone deacetylase HDA705 reduces the acetylation of OsECT3, a plant m^6^A reader protein, at lysine 471 (K471) (Ma et al., 2025). This deacetylation enhances the m^6^A-binding activity of OsECT3, which then binds to and stabilizes the transcripts of *COLD1* and other cold-response-related genes, thereby enhancing cold tolerance (Ma et al., 2025). Another histone deacetylase, OsHDA716, regulates cold stress responses by mediating the deacetylation of OsbZIP46. OsbZIP46 is a transcription factor that controls the expression of *COLD1* and *OsDREB1A* (Kamble, 2024; Sun et al., 2024). Under normal temperatures, OsbZIP46 deacetylated by OsHDA716 is prone to proteasomal degradation; simultaneously, OsHDA716 represses the expression of OsbZIP46 target genes through histone deacetylation of their promoters. Under cold stress, cold signals induce the degradation of OsHDA716, which prevents the deacetylation of OsbZIP46 at K263, K281, and K294. The accumulated acetylated OsbZIP46 then activates the transcription of *COLD1* and *OsDREB1A*, leading to enhanced cold tolerance (Sun et al., 2024). Additionally, OsDREB2B regulates *COLD1* expression by forming a complex with OsSRO1c, a protein with intrinsic liquid-liquid phase separation properties, in the nucleus (Hu et al., 2024). The phase separation of the OsSRO1c-OsDREB2B complex dynamically responds to low temperatures and enhances the transcription of *COLD1* and other COR genes (Hu et al., 2024). Given the central role of COLD1 in rice cold stress responses, it is anticipated that more transcription factors and regulatory mechanisms governing *COLD1* expression will be discovered in future studies.

## 3 OsbHLH002/OsICE1-OsDREBs-COR core transcriptional module

### 3.1 Regulation of OsbHLH002/OsICE1

**Figure 3:**
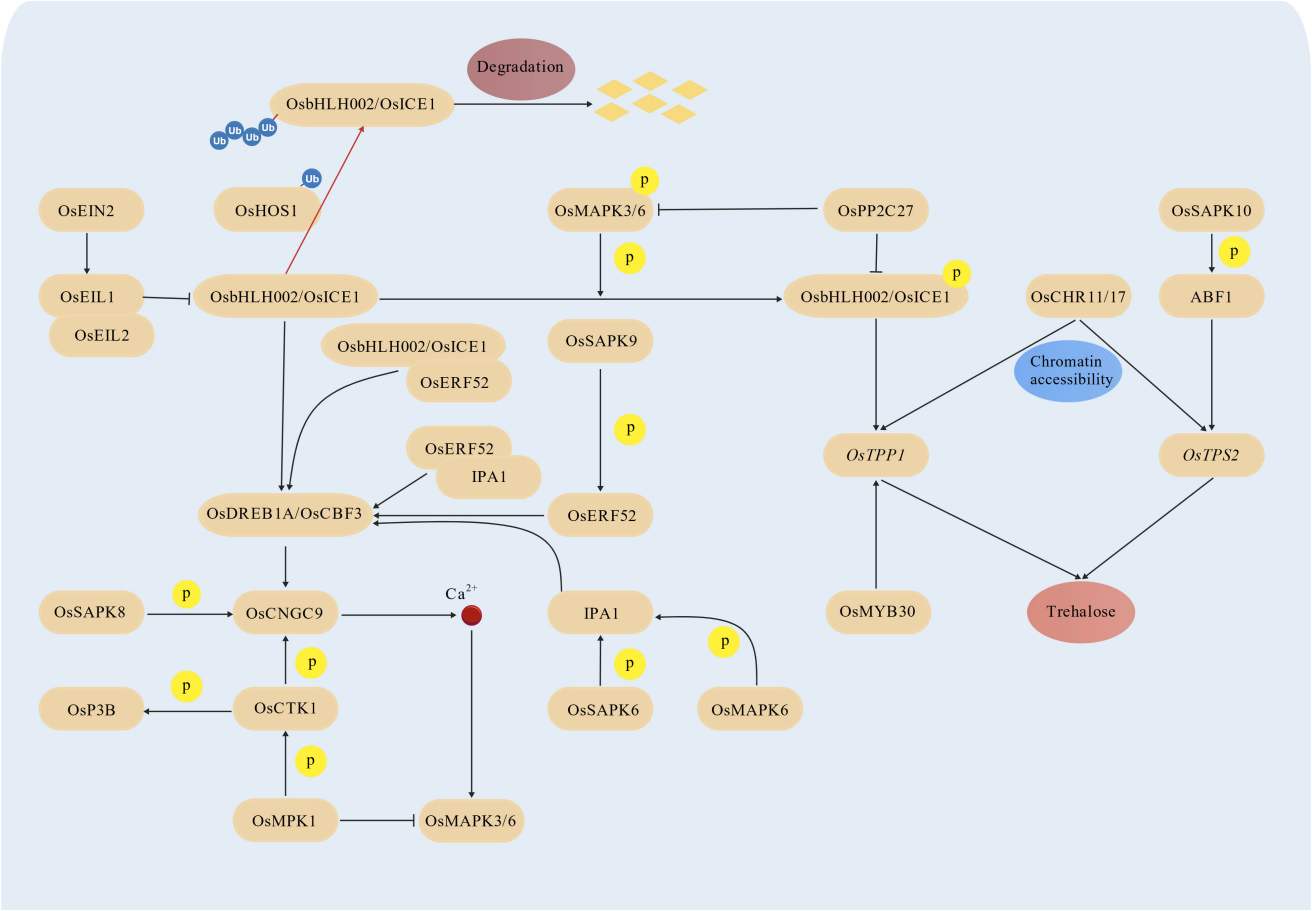
OsbHLH002/OsICE1-OsDREBs-COR core transcriptional modules in rice.

OsbHLH002/OsICE1 (INDUCER OF CBF EXPRESSION 1) is a master transcription factor in the rice cold stress signaling pathway (**Figure 3**). Unlike its homolog in Arabidopsis (*AtICE1*), *OsbHLH002*/*OsICE1* expression is minimally affected by cold stress (Chinnusamy et al., 2003; Nakamura et al., 2011), its function is primarily regulated at the post-transcriptional and post-translational levels. It was reported that the expression of *OsbHLH002*/*OsICE1* is negatively regulated by the OsEIN2-OsEIL1/2 module, which is a key component of the ethylene signaling pathway (Zhai et al., 2024). Specifically, OsEIL1 and OsEIL2, two master regulators of ethylene signaling, form a heterodimer and directly bind to the promoter of *OsbHLH002*/*OsICE1*, thereby synergistically suppressing its transcription (Zhai et al., 2024).

Post-translational modifications (PTMs) play a critical role in regulating the accumulation and activity of OsbHLH002/OsICE1, and thus mediate cold stress signaling (**Figure 3**). The E3 ubiquitin ligase OsHOS1 (HIGH EXPRESSION OF OSMOTICALLY RESPONSIVE GENES 1) mediates the ubiquitination of OsbHLH002/OsICE1, marking it for degradation by the 26S proteasome (Lourenco et al., 2013). Under cold stress, OsMAPK3 and OsMAPK6 interact with and phosphorylate OsbHLH002/OsICE1, which prevents its ubiquitination by OsHOS1 and promotes its accumulation (Zhang et al., 2017; Liu et al., 2023a). The accumulated phosphorylated OsbHLH002/OsICE1 directly binds to the promoter of *OsTPP1* (trehalose-6-phosphate phosphatase 1) and activates its expression, leading to increased trehalose biosynthesis and cold tolerance (Ge et al., 2008; Zhang et al., 2017). OsPP2C27, a type 2C protein phosphatase, functions as a negative regulator of the OsMAPK3/6-OsbHLH002/OsICE1-OsTPP1 pathway (Xia et al., 2021). It directly dephosphorylates both OsMAPK3/6 and OsbHLH002/OsICE1, thereby attenuating the activity of this pathway (Xia et al., 2021). This negative regulation is essential for preventing the sustained activation of cold stress responses under cold conditions, which could otherwise compromise normal plant growth and development. Together, OsbHLH002/OsICE1 orchestrates multiple signaling pathways through various post-translation modifications, including ubiquitination, phosphorylation, and dephosphorylation, in response to cold stress in rice.

### 3.2 Multi-layered regulation of *OsDREB*s

In addition to *OsTPP1*, *OsDREB*s are key direct targets of OsbHLH002/OsICE1, forming the core OsbHLH002/OsICE1-OsDREBs-COR regulatory axis in the rice cold stress signaling pathway (**Figure 3**) (Zhang et al., 2019b). Other transcription factors also contribute to the regulation of *OsDREB*s. OsERF52, an AP2/ERF family member, and IPA1, a SPL family member, directly bind to the promoters of *OsDREB*s and regulate their expression (Jia et al., 2022; Xu et al., 2024). Furthermore, OsSAPK9, a sucrose non-fermenting 1-related protein kinase 2, phosphorylates OsERF52 at serine 261 (S261), which enhances the protein stability of OsERF52 and strengthens its interaction with IPA1 and OsbHLH002/OsICE1. These interactions synergistically activate the transcription of *OsDREB*s, thereby initiating cold stress responses (Xu et al., 2024). Similar to OsERF52, IPA1 is phosphorylated by a kinase, OsSAPK6, and the phosphorylation increases its protein stability. Genetic studies have confirmed that serine 213 (S213) is a critical phosphorylation site mediating the function of IPA1 in rice cold stress responses (Jia et al., 2022). Additionally, OsMAPK6 phosphorylates IPA1, further modulating its activity and influencing cold stress tolerance in rice (Liu et al., 2023a).

### 3.3 Regulation of COR genes: a case study of *OsCNGC9*

Among the COR genes directly targeted by *OsDREB*s, *OsCNGC9*, a cyclic nucleotide-gated channel, is a well-studied target of OsDREB1A. OsDREB1A directly activates the expression of *OsCNGC9* (**Figure 3**) (Wang et al., 2021). Recent studies have shown that OsCNGC9 is phosphorylated by two kinases, OsSAPK8 and OsCTK1 (Wang et al., 2021; Wu et al., 2024). Specifically, the phosphorylation of OsCNGC9 at serine 645 (S645) by OsSAPK8 significantly enhances its channel activity, promoting cytosolic Ca²^+^ influx under cold stress and amplifying the cold signal (Wang et al., 2021). Beyond OsCNGC9, OsCTK1 phosphorylates other substrates involved in cold tolerance, including the acidic ribosomal protein OsP3B and the dual-specific mitogen-activated protein kinase phosphatase OsMKP1 (Wu et al., 2024). Notably, OsMKP1 negatively regulates the kinase activity of OsMAPK3/6 that can phosphorylate OsbHLH002/OsICE1 and IPA1 (Zhang et al., 2017; Liu et al., 2023a), forming a negative feedback loop that fine-tunes the MAPK signaling cascade during cold stress responses.

### 3.4 Regulation of trehalose biosynthesis, an outcome of OsbHLH002/OsICE1-OsDREBs-COR module

Cold stress-induced biosynthesis and accumulation of osmolytes play a crucial role in osmotic adjustment, counteracting the osmotic pressure imposed by cold stress (Li et al., 2022). Among these osmolytes, trehalose is an important outcome of OsbHLH002/OsICE1-OsDREBs-COR core module, and enhances rice cold tolerance (**Figure 3**). Its biosynthesis is tightly regulated by two critical enzymes, trehalose-6-phosphate synthase (TPS) and trehalose-6-phosphate phosphatase (TPP) (Lunn et al., 2014; Eh et al., 2025). The genes encoding these enzymes, OsTPS2 (TPS) and OsTPP1 (TPP), are subject to complex transcriptional regulation in response to cold stress (Lunn et al., 2014; Eh et al., 2025). *OsTPP1* is not only regulated by OsbHLH002/OsICE1 but also by OsMYB30 (Yu et al., 2024). *OsTPS2* is regulated by ABF1, a bZIP family transcription factor, which is induced by cold stress (Shu et al., 2023). OsSAPK10 phosphorylates ABF1, which strengthens its DNA-binding ability to the cis-acting elements in the *OsTPS2* promoter, thereby enhancing *OsTPS2* transcription and trehalose biosynthesis (Shu et al., 2023). Chromatin accessibility also plays a crucial role in regulating the expression of *OsTPS2* and *OsTPP1* under cold stress. The imitation switch (ISWI)-type chromatin remodeling factors OsCHR11 and OsCHR17 physically associate with the promoters of *OsTPS2* and *OsTPP1*, increasing chromatin accessibility and ensuring the full induction of these genes under cold stress conditions (Li et al., 2024a).

## 4 Other transcriptional factor pathways in rice cold stress response

**Figure 4:**
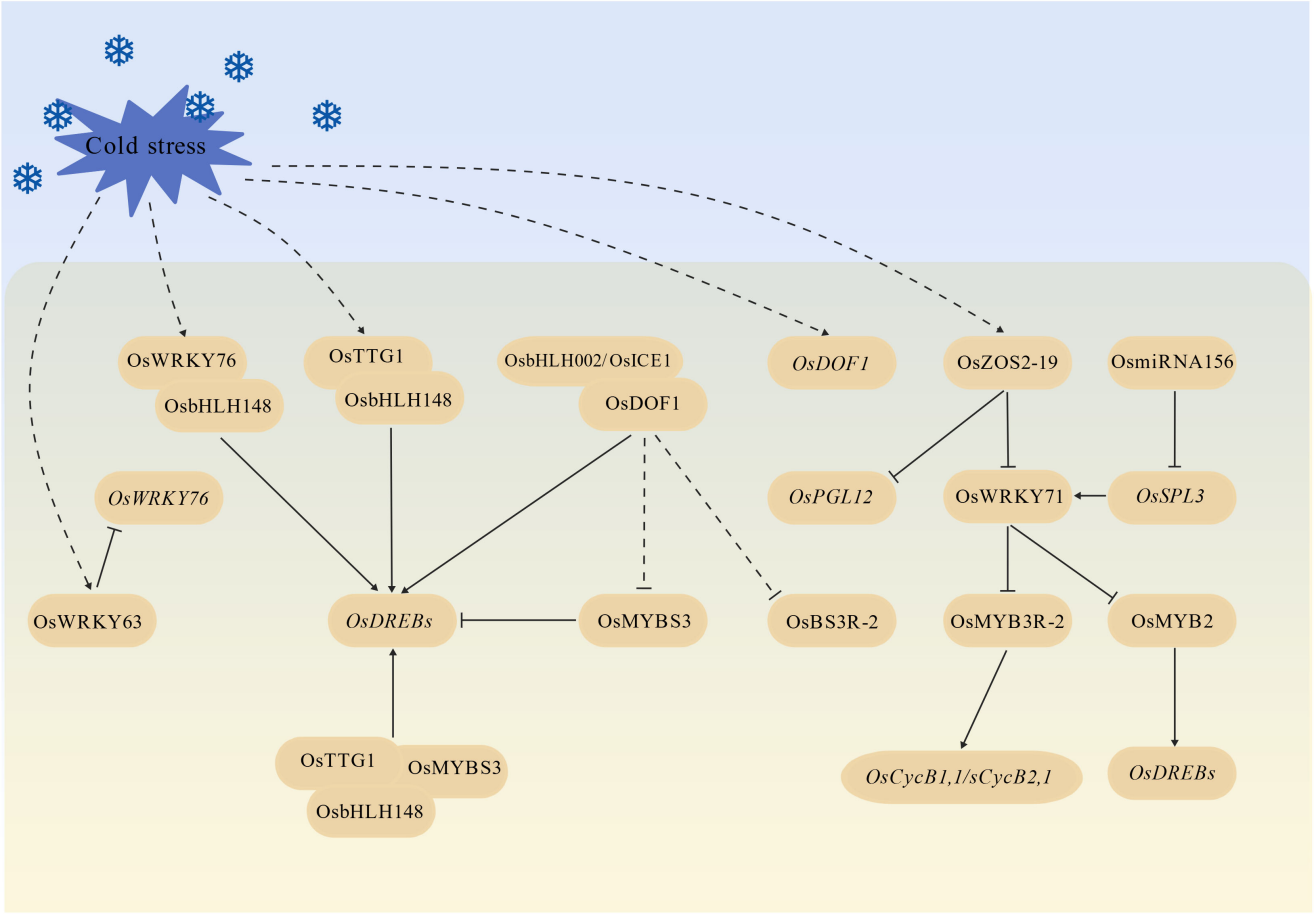
Other transcriptional factor pathways in rice cold stress response.

Beyond the OsbHLH002/OsICE1-OsDREBs-COR core module, a diverse array of other transcription factors form a complex regulatory network to modulate rice cold responses (**Figure 4**). *OsWRKY63* is induced by cold stress and acts as a negative regulator of rice cold tolerance (Zhang et al., 2022). Concurrently, OsWRKY63 directly represses the expression of *OsWRKY76*, a positive cold tolerance regulator. OsWRKY76 interacts with OsbHLH148 to directly activate the transcription of *OsDREB1B* (Zhang et al., 2022). OsbHLH148 also forms a MBW (MYB-bHLH-WD40) complex with MYBS3 and OsTTG1, a WD40 repeat protein, which further enhances the expression of *OsDREB1*s (Zhu et al., 2024). However, conflicting reports suggest that MYBS3 can also act as a negative regulator of *OsDREB*1s (Su et al., 2010), indicating that its function may be context-dependent (e.g., varying with the rice growth stage or stress intensity).

OsDOF1, a DOF family transcription factor, is induced by cold stress and positively regulates cold tolerance (Liu et al., 2021, 2023). It directly interacts with OsbHLH002/OsICE1 to enhance the expression of *OsDREB*s, while simultaneously repressing the expression of two cold-responsive transcription factors, *OsMYBS3* and *OsBS3R-2* (Liu et al., 2023b). OsZOS2–19 is a C2H2 zinc finger protein. It is upregulated by cold stress and functions as a transcriptional repressor (Zhang et al., 2025). It suppresses the expression of COR genes such as *OsPGL12* (involved in osmotic stress responses) and *OsWRKY71* (involved in ROS scavenging) (Kim et al., 2016; Chen et al., 2018), thereby attenuating cold tolerance (Zhang et al., 2025). Although *OsWRKY71* is negatively regulated by OsZOS2-19, it is also positively regulated by OsSPL3 that is a SPL family member targeted by OsmiR156 (Zhou and Tang, 2019). Further studies have revealed that *OsMYB3R-2* and *OsMYB2* are the downstream targets of OsWRKY71. Both *OsMYB3R-2* and *OsMYB2* are negatively regulated by OsWRKY71. OsMYB3R-2 regulates the expression of cell cycle-related genes, such as *OsCycB1;1* and *OsCycB2;1*, which are hypothesized to mediate rice growth under cold stress by maintaining cell division. OsMYB2 directly binds to the promoters of *OsDREB*s and activates their expression to modulate rice cold response (Zhou and Tang, 2019). Collectively, these findings demonstrate that beyond the OsbHLH002/OsICE1-OsDREBs-COR module, a highly complex transcriptional network regulates rice cold stress responses.

## 5 Membrane lipid remodeling in rice cold stress response

As frontline responders to cold stress, plant membranes are among the primary targets of cold-induced damage. To sustain normal physiological function, plant membranes must maintain their integrity and fluidity under cold stress (Shomo et al., 2024). Increasement of polyunsaturated fatty acid contents is a proviral strategy to maintain integrity and fluidity of membrane under cold stress (Nozawa, 2011). Tovuu et al. uncovered that OsFAD8, a cold-inducible ω-3 fatty acid desaturase (FAD), plays an important role in C18 fatty acid (FA) unsaturation under cold stress. Compared to wild-type seedlings, the *osfad8* mutant exhibited reduced lower levels of 18:3 FA and a lower 18:3/18:2 ratio, accompanied by decreased concentrations of key membrane lipid including monogalactosyl diacylglycerol (MGDG), digalactosyl diacylglycerol (DGDG), sulfoquinovosyl diacylglycerol (SQDG), and phosphatidyl glycerol (PG). Further analysis confirmed that *osfad8* mutant displayed a significant reduction in membrane fluidity and enhanced sensitive to short-term low temperature stress (Tovuu et al., 2016). It was demonstrated that OsTIL1 lipocalin, which can bind to and transport a variety of lipids, plays an important positive role in FADs-mediated glycerolipid remodeling under cold stress (Ji et al., 2024). Under cold stress, *OsTIL1* overexpression lines exhibited higher 18:3 FA contents, elevated 18:3/18:2 and (18:2 + 18:3)/18:1 ratios, as well as increased accumulation of 18:3-containing glycerolipids under cold stress, including galactolipids (MGDG and DGDG) and phospholipids [PG, phosphatidylcholine (PC), phosphatidylethanolamine (PE), phosphatidylserine (PS), and phosphatidylinositol (PI)]. Meanwhile, *OsTIL1* overexpression lines displayed enhanced the transcriptional levels and enzyme abundances of four FADs, namely OsFAD3-1, OsFAD3-2, OsFAD7, and OsFAD8, under cold stress conditions (Ji et al., 2024). *CTB6*, which is mainly expressed in tapetum and young microspores of the anther, encodes a lipid transfer protein possessing lipid-binding ability, and positively affects the triacylglycerol (18:0_18:0_18:3) content in anthers to regulate cold tolerance (Gao et al., 2025). Additionally, CTB6 interacts with catalases to maintain their stability, thereby scavenging ROS accumulation and facilitating tapetum development under cold stress conditions (Gao et al., 2025). *OsKASI-2* encodes a chloroplast-localized KASI enzyme, which is mainly expressed in leaves and anthers and strongly induced by cold stress (Zhang et al., 2024). Membrane lipid analysis of *oskasI-2* mutant and *OsKASI-2* overexpression lines revealed that *OsKASI-2* positively regulate the content of unsaturation FAs (C16:1 and C20:5). The exogenous application assay indicated that C16:1 and C20:5 contents contribute to *OsKASI-2*-regulated cold tolerance in rice (Zhang et al., 2024). As an early cold-inducible phospholipase, OsPLDα1 can hydrolyzed phosphatidylcholine, a central glycerolipid, to produce the signal molecular phosphatidic acid. Genetic studies indicated that *ospld*α*1* mutant is more susceptible to cold stress (Huo et al., 2016). Transcriptional regulation analysis showed that the expression of OsDREB1s is inhibited in *ospld*α*1* mutant and OsDREB1A can directly regulate the expression of *OsPLDα1*. These lines of evidences suggested that OsPLDα1 serves a pivotal role in cold signal transduction in rice, rather than merely functioning in membrane lipid remodeling (Huo et al., 2016). Together, these studies demonstrated that membrane lipid remodeling is critical for rice tolerance in response to cold stress.

## 6 Phytohormones in rice cold stress response

**Figure 5:**
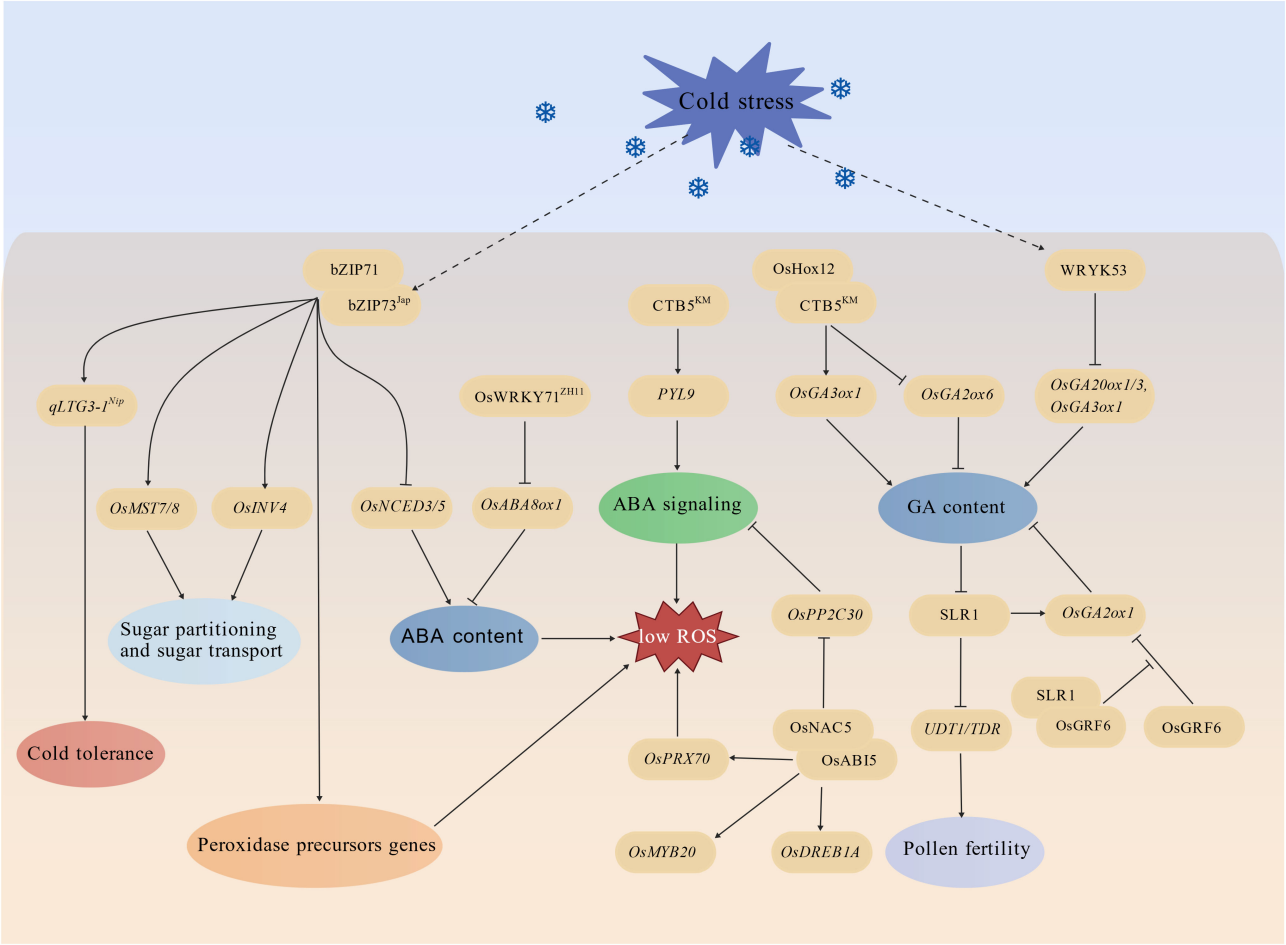
ABA and GA modulation in rice cold stress response.

Within the intricate regulatory networks that govern plants’ responses to cold stress, phytohormones act as core signaling molecules. They play pivotal roles in coordinating physiological and molecular adaptations. Notably, the mechanisms underlying rice’s cold stress responses are tightly intertwined with the synergistic regulation of phytohormones. Among these, abscisic acid (ABA) and gibberellic acid (GA) have emerged as the most extensively investigated due to their distinct yet interconnected roles in mediating cold tolerance (**Figure 5**).

### 6.1 Gibberellic acid

It was validated that GA biosynthesis and signaling pathway are implicated in rice cold response. GA exhibits unique and indispensable regulatory functions, primarily centered on alleviating cold-induced growth inhibition and coordinating the balance between growth and stress tolerance. OsWRKY53 is induced by cold stress. It directly suppresses the expression of GA biosynthetic genes, such as *GA20ox1*, *GA20ox3*, and *GA3ox1*, leading to reduced GA biosynthesis and lower intracellular GA levels in anthers (Fang et al., 2017; Tang et al., 2022). The decrease in GA content promotes the accumulation of DELLA protein SLR1 (SLENDER RICE 1), a negative regulator of GA signaling. SLR1 interacts with and suppresses the transcriptional activity of UDT1 (UNDEVELOPED TAPETUM 1) and TDR (TAPETUM DEGENERATION RETARDATION), which is essential for tapetum development and pollen maturation (Tang et al., 2022). This interaction results in pollen infertility under cold stress, a major cause of yield loss in rice. SLR1 also interacts with OsGRF6 (GROWTH-REGULATING FACTOR 6). OsGRF6 is a transcription factor that promotes GA biosynthesis by repressing the expression of *OsGA2ox1*, a GA catabolic gene, to respond to cold stress in rice (Li et al., 2021). Under normal temperatures, OsGRF6 represses *OsGA2ox1*, thereby maintaining active GA levels to support plant growth. Under cold stress, SLR1 directly promotes *OsGA2ox1* expression. Meanwhile, SLR1 interacts with OsGRF6, relieving the repression of *OsGA2ox1*. This leads to increased GA catabolism, reduced active GA levels, and enhanced cold tolerance in order to balance growth and stress responses (Li et al., 2021). Other transcription factors also regulate GA metabolism in response to cold stress. CTB5, a HD-Zip family member, interacts with OsHox12, another HD-Zip family member, to activate the expression of the GA biosynthetic gene *OsGA3ox1* and inhibit the expression of *OsGA2ox6*, a GA catabolic gene (Guo et al., 2025). This regulation promotes GA accumulation in anthers, facilitating tapetum development and pollen fertility under cold stress (Guo et al., 2025). Additionally, CTB5 directly binds to the promoter of *PYL9*, an ABA receptor-encoding gene, enhancing cold tolerance at the seedling stage by reducing ROS accumulation. This regulation mechanism highlights the crosstalk between GA and ABA signaling in cold responses (Guo et al., 2025). Evolutionary studies have revealed that the favorable *CTB5^KM^* allele, a natural variant of *CTB5*, was selected during the cold acclimation of *japonica* rice adapted to plateau habitats in Yunnan Province, China, underscoring the role of natural variation in shaping cold tolerance in rice (Guo et al., 2025).

### 6.2 Abscisic acid

Unlike gibberellic acid (GA) which focuses on growth recovery, ABA primarily functions as a key regulator of cold stress defense, orchestrating a series of physiological and molecular responses to enhance rice tolerance to low temperatures. Direct evidence indicating the important role of ABA in rice cold response is null mutants of ABA biosynthetic genes *OsNCED4* and *OsNCED5* display reduced cold tolerance (Xiang et al., 2024, 2025). OsNAC5 is a cold-induced NAC family transcription factor. It modulates rice cold tolerance by fine-tuning the ABA signaling pathway (Takasaki et al., 2010; Li et al., 2024b). OsNAC5 directly activates the expression of *OsABI5* (*ABA INSENSITIVE 5*), which is a key transcription factor in ABA signaling pathway. Meanwhile, it physically interacts with OsABI5 to enhance its protein stability (Li et al., 2024b). *In vivo* and *in vitro* assays have shown that OsABI5 directly regulates the expression of three classes of genes. First, it targets *OsPP2C30* that encode a type 2C protein phosphatase and negatively regulates ABA signaling (Kim et al., 2011). Second, it regulates *OsPRX70*, which encodes a ROS-scavenging peroxidase that reduces cold-induced oxidative damage. Third, it controls COR genes including *OsDREB1A* and *OsMYB20*, integrating ABA signaling with the core cold response pathway (Li et al., 2024b).

The bZIP family transcription factors also play an important role in rice cold tolerance by mediating ABA biosynthesis. bZIP73^jap^, a *japonica*-specific variant of bZIP73, and bZIP71 form a heterodimer that regulates cold tolerance through four distinct signaling pathways. First, the bZIP73^jap^:bZIP71 heterodimer inhibits the expression of *OsNCED3* and *OsNCED5*, reducing ABA levels and preventing excessive growth inhibition under cold stress (Liu et al., 2018a; Li et al., 2025). Second, the bZIP73^jap^:bZIP71 heterodimer targets to the promoters of four peroxidase (POX) precursor genes (*LOC_Os01g22249*, *LOC_Os03g02920*, *LOC_Os03g32050*, and *LOC_Os04g59210*) and activates their expression, thereby reducing the ROS accumulation. Third, it directly targets the promoters of sugar transport-related genes (*OsMST7*, *OsMST8*, and *OsINV4*), enhancing soluble sugar transport and partitioning. Soluble sugars act as compatible solutes and cryoprotectants, protecting cells from cold-induced damage (Bolouri-Moghaddam et al., 2010). Fourth, the bZIP73^jap^:bZIP71 heterodimer also activates the expression of *qLTG3-1*^Nip^, a natural variant of *qLTG3–1* from *Nipponbare* (Fujino et al., 2008). qLTG3-1^Nip^ interacts with bZIP73^jap^ and enhances the transcriptional activity of bZIP73^jap^, forming a positive feedback loop that amplifies cold tolerance (Liu et al., 2019). Additionally, OsWRKY71^ZH11^, a variant of OsWRKY71 from Zhonghua 11, directly suppresses the expression of *OsABA8ox1*, an ABA catabolic gene, under cold stress, increasing ABA levels and enhancing cold tolerance, which further highlights the role of ABA metabolism in rice cold responses (Li et al., 2025).

In recent years, many findings have indicated that many other phytohormones, such as auxin, jasmonate, salicylic acid, ethylene, and brassinosteroid are also involved in rice cold response (Mao et al., 2019; Jiang et al, 2020; Cheng et al., 2024; Cui et al., 2025; Xu et al., 2025). However, the molecular mechanisms remain to be explored.

## 7 Perspectives

Despite significant advances in unraveling the molecular framework of rice cold stress responses, several critical knowledge gaps remain, and targeted exploration in the following areas will further deepen our understanding and accelerate the translation of basic research into cold-tolerant rice breeding:

1. Deciphering the functional mechanisms and signaling crosstalk of 2’,3’-cAMP

As a newly identified secondary messenger in rice cold signaling, 2’,3’-cAMP’s regulatory role is far from fully understood (Luo et al., 2024). Current studies only confirm its cold-induced accumulation and association with COR gene activation (e.g., OsDREBs), but key questions persist: What are the direct effectors of 2’,3’-cAMP? Do proteins such as cyclic nucleotide-binding domains (CNBDs)-containing proteins mediate its downstream signaling, and how do these effectors interact with other signaling components? More importantly, the crosstalk between 2’,3’-cAMP and Ca²^+^, the two core secondary messengers, requires systematic dissection.

2. Mining and utilizing universal cold-tolerant genes with minimal yield penalty

A major challenge in cold-tolerance breeding is balancing stress resistance with yield performance—many currently identified cold-tolerant genes (e.g., overexpression of *OsDREB1A*, *OsDREB1B*, and *OsDREB1C*) enhance cold resistance but often cause growth retardation or yield loss under normal conditions (Ito et al., 2006). Future efforts could prioritize two directions: First, mining tissue-specific or stress-inducible cold-tolerant genes from rice germplasm (e.g., wild rice *O. rufipogon*, cold-adapted *japonica* landraces). For example, genes specifically expressed in anthers (to protect pollen fertility under cold) or roots (to maintain water/nutrient uptake) could improve targeted cold tolerance without affecting vegetative growth or grain filling. Second, leveraging natural variants of key regulatory genes with favorable pleiotropic effects. For instance, the *CTB5^KM^* allele (selected in Yunnan plateau *japonica*) enhances cold tolerance while preserving yield, and similar variants in genes like *COLD1* also exist in diverse rice accessions (Guo et al., 2025; Ma et al., 2015). Additionally, genome-editing tools (e.g., CRISPR-Cas9) can be used to modify cis-acting elements of cold-responsive genes (e.g., adding stress-specific promoters) to restrict their expression to stress conditions, minimizing yield trade-offs.

3. Expanding the exploration of epigenetic regulation and non-coding RNA-mediated cold responses

Beyond transcriptional and post-translational regulation, epigenetic modifications (e.g., DNA methylation, histone modification, m^6^A RNA methylation) and non-coding RNAs (ncRNAs, e.g., miRNAs, lncRNAs, and circRNAs) are emerging as critical regulators of cold stress responses. For example, the hypomethylation of *ACT1* promoter is heritable and contributes to the acquisition of adaptive cold tolerance in rice (Song et al., 2025). However, it remains unclear whether hypomethylation (or methylation changes) at other genomic loci, which follows a similar pattern, contributes to rice heritable cold tolerance. HDA705 modulates *COLD1* expression via m^6^A-dependent RNA stabilization (Ma et al., 2025), but the role of other epigenetic modifiers (e.g., DNA methyltransferases, histone methyltransferases) in cold signaling remains largely unknown. Similarly, ncRNAs such as OsmiR156 have been implicated in cold tolerance (Zhou and Tang, 2019), but most cold-responsive ncRNAs and their targets await identification. Future studies should combine multi-omics approaches (e.g., epigenomics, transcriptomics, degradomics) to systematically map the epigenetic and ncRNA regulatory networks of cold stress, uncovering new layers of control in rice cold adaptation.

4. Protein degradation in rice under cold stress merits additional scientific inquiry

Beyond the transcriptional regulation and signaling transduction pathways, protein degradation plays an indispensable role in maintaining protein quality control during plant development as well as in responses to abiotic and biotic stresses. As one of the core protein degradation system, the ubiquitin-proteasome system has been well-demonstrated to exert a pivotal function in rice cold stress adaption. For instance, multiple ubiquitin E3 ligases have been identified as key regulators involved in rice cold stress response (Lourenco et al., 2013; Byun et al., 2017; Zhang et al., 2017; Cui et al., 2018). However, the degradation mechanisms of numerous other critical proteins in cold stress signaling pathway remain elusive. Notably, the functional roles of autophagy, the other core protein degradation system, including selective autophagy in mediating rice cold stress responses is still far from being fully elucidated.

5. Single-cell and spatial multi-omics have emerged as robust, cutting-edge approaches to decipher the molecular basis of cold stress acclimation in rice

Single-cell and spatial multi-omics have been widely applied in plant science, dramatically advancing our understanding of cellular heterogeneity, and gene expression patterns, and metabolite dynamics within plant tissues (Giacomello, 2021; Yu et al., 2023; Serrano et al., 2024). Therefore, these powerful cutting-edge techniques hold great promise for illuminating the molecular mechanisms underlying rice’s response to cold stress at the single-cell resolution.

In summary, addressing these gaps will not only advance our fundamental understanding of plant cold stress biology but also provide novel genetic targets and strategies for developing cold-tolerant rice varieties, ultimately safeguarding global rice production under the threat of climate change.
